# Active enhancer positions can be accurately predicted from chromatin marks and collective sequence motif data

**DOI:** 10.1186/1752-0509-7-S6-S16

**Published:** 2013-12-13

**Authors:** Agnieszka Podsiadło, Mariusz Wrzesień, Wiesław Paja, Witold Rudnicki, Bartek Wilczyński

**Affiliations:** 1 Institute of Informatics, University of Warsaw, Banacha 2, 02-097 Warsaw, Poland; 2 University of Information Technology and Management in Rzeszów, Sucharskiego 2, 35-225 Rzeszów, Poland; 3 Interdisciplinary Centre for Mathematical and Computational Modelling, University of Warsaw, Pawińskiego 5A, 02-106 Warsaw, Poland

## Abstract

**Background:**

Transcriptional regulation in multi-cellular organisms is a complex process involving multiple modular regulatory elements for each gene. Building whole-genome models of transcriptional networks requires mapping all relevant enhancers and then linking them to target genes. Previous methods of enhancer identification based either on sequence information or on epigenetic marks have different limitations stemming from incompleteness of each of these datasets taken separately.

**Results:**

In this work we present a new approach for discovery of regulatory elements based on the combination of sequence motifs and epigenetic marks measured with ChIP-Seq. Our method uses supervised learning approaches to train a model describing the dependence of enhancer activity on sequence features and histone marks. Our results indicate that using combination of features provides superior results to previous approaches based on either one of the datasets. While histone modifications remain the dominant feature for accurate predictions, the models based on sequence motifs have advantages in their general applicability to different tissues. Additionally, we assess the relevance of different sequence motifs in prediction accuracy showing that even tissue-specific enhancer activity depends on multiple motifs.

**Conclusions:**

Based on our results, we conclude that it is worthwhile to include sequence motif data into computational approaches to active enhancer prediction and also that classifiers trained on a specific set ofenhancers can generalize with significant accuracy beyond the training set.

## Background

Transcriptional regulation in development is a complex biological process that is absolutely essential for the existence of multi-cellular organisms, especially in the metazoa kingdom. While the main principles of transcriptional regulation on the molecular level have been discovered in 1960s [1], and we do have relatively complete pictures of transcriptional regulation in single-cell model organisms such as *E. coli *[2] or *S. cerevisiae *[3], we still don't have a complete map of developmental regulation for even a singlemulti-cellular organism.

One feature that clearly differentiates multi-cellular species from simpler organisms is the modularity of regulatory elements. In microbial systems, transcription factors bind directly to gene promoters and modulate gene activity via direct repression or activation. In metazoan systems, it is more typical for a gene to have multiple regulatory elements, attracting collections of transcription factors and regulating target gene expression in a combinatorial fashion sometimes over large genomic distances. Important class of regulatory elements are enhancers: discrete DNA elements, able to enhance expression of their target genes in a tissue specific fashion. Since enhancer activity can be tested by creating transgenic reporter assays, they are able to act independently of each other and cannot require any specific chromosomal context. This modular structure of regulatory sequences, particularly evident in developmental regulation [4], makes it difficult to build comprehensive models of transcriptional networks. In order to make it more tractable, the task of building global models can be broken down into two distinct sub-problems: identification of all relevant regulatory sequences and linking them with respective target genes. Recently, we have shown [5] that in cases where we have a biological model with an experimentally verified map of enhancer elements, the second problem can be tackled with a probabilistic model giving high accuracy of predictions of both target genes and their tissue-specific expression. However, the first problem of finding the positions of all enhancers still poses a major challenge for the bioinformatics community.

Historically, there have been two main bioinformatical approaches to enhancer discovery. Firstly, people have observed that clustering of transcription factor binding sites is an indication of enhancer activity [6]and secondly, it has been shown in multiple cases that many functional enhancers are evolutionarily more conserved than other non-coding sequences in a genome [7]. Soon, these two observations were used together to give rise to multiple methods using evolutionary conservation and motif enrichment to find functional regulatory elements [8,9].

While methods based solely on the sequence information have achieved significant enrichment for true enhancers among their predictions, they are still prone to errors. On one hand, many of predicted enhancers are not functional because of contextual factors such as chromatin conformation [10] leading to false positive predictions. On the other hand, enhancers responsible to species-specific or recently evolved features are bound to fail the evolutionary conservation filters leading to false negative predictions [11]. More recently, due to development of methods for experimental measurements of histone marks and other epigenetic features [12] it has become standard to identify regulatory regions *en masse *by ChIP-Seq experiments on such factors as H3k4me1 [13] or p300 [14]. Major experimental efforts such as ENCODE [15] are now underway to map multiple chromatin marks in as many conditions as possible, leading to more direct epigenetic maps of the genome. While these measurements are more directly assaying functionality of regulatory elements, they are, unfortunately, not a perfect solution. In particular, in a recent study [16], we were able to show that not only is the activity of enhancers "encoded" in multiple marks, but the epigenetic patterns associated with enhancer activity are non-additive, making it more complex to recover truly active regions.

In this work we attempt to combine the strengths of both sequence-based and chromatin-based methodsfor enhancer prediction while avoiding the difficulties associated with each of these approaches. In the following sections we will describe the method itself and present the results obtained with this approach on several datasets consisting of different regulatory elements in the *Drosophila melanogaster *model organism.

## Results and discussion

### Predicting enhancer activity from histone modifications

**Figure 1:**
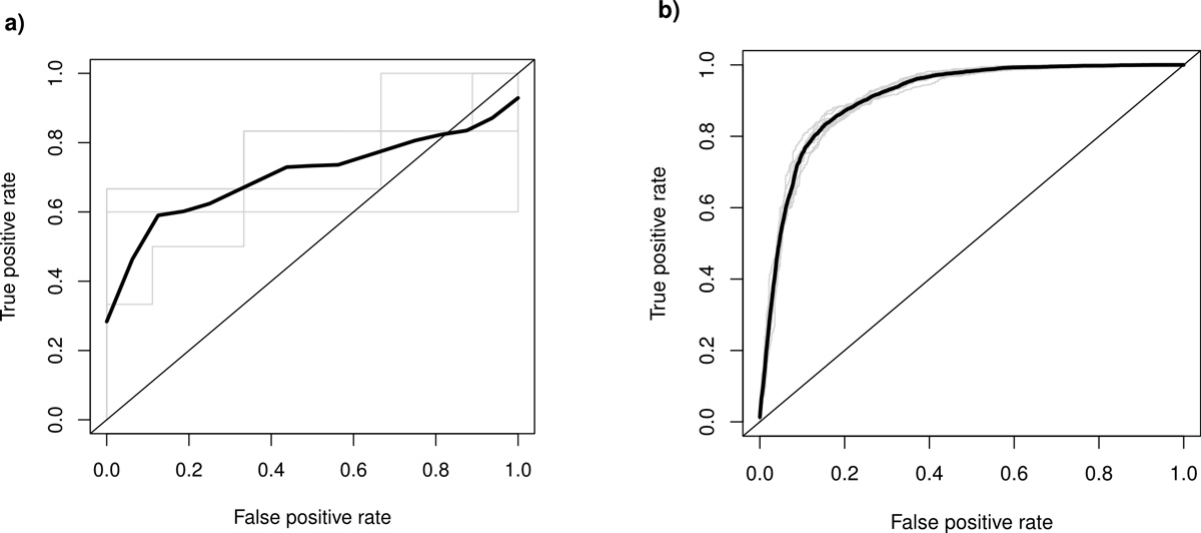
**Comparison of prediction quality from histone marks**. Difference in prediction quality achieved with BNFinder on epigenetic features for dataset of different sizes: 64 examples from [16] - AUC of0.75 on average (a) and 8008 examples from [17] - AUC of 0.93 on average (b). Both experiments are reported for cross-validated training.

Our first attempt was to reproduce results from a recent paper by Bonn et al. [16], where we used a Bayesian network classifier to predict enhancers from chromatin features (6 histone modifications, PolII occupancy and Mef2 binding). While we were able to obtain a similar prediction accuracy (80%), due to the small size of the training set, the variability on prediction quality between cross-validation folds was very high (see Figure 1). For this reason, we have re-computed the epigenetic features for a larger set of putative CRMs compiled by Zinzen *et al*. [17] from Chip-chip experiments. This dataset (see Table S3, Additional file 3) is much larger (8008 putative enhancers and 8008 random regions in contrast to 62 verified enhancers), however it is not fully experimentally validated. Assuming that the validation results from the work of Zinzen [17] can be extrapolated to the whole dataset, we expect not more than 5% of errors in this dataset (see Methods for details). In Figure 1b we can see that the Bayesian network classifier performs much better on the larger dataset (AUC of 0.93 as opposed to 0.75 for the smaller dataset). As the larger dataset proves to be much better for classifier training, we have focused on it in our further analysis.

### Using sequence motifs to improve predictions

While features based on histone modifications contain enough information to obtain good and reproducible classifier training, we wanted to verify if the same can be predicted from the DNA sequence of the respective sequences and to what extent the sequence motif information is redundant with the epigenetic component. To this end we have used all 125 insect related transcription factor binding site motifs deposited in the publicly available JASPAR database [18]. Even though they represent less than half of the estimated total number of transcription factors in the Drosophila genome, they represent all major classes of DNA-binding domains. Based on our earlier results [9], we assumed that this motif set should allow us to make reasonable predictions of enhancers based only on the motif occurrences. We extracted the DNA sequences of all positive and negative examples and computed the thermodynamical binding energy score (TRAP) [19] for each motif-sequence pair. This gave us a much larger feature set (125 features) in comparison with the epigenetic marks.

Due to the high complexity of Bayesian Network reconstruction, BNFinder is not recommended for analysis of datasets with large sets of feature. For this reason, we have tested two popular general classification methods: Support Vector Machines (SVMs) [20] and Random Forests (RFs) [21]. In order to assess the quality of motif features and its redundancy with the epigenetic marks we have trained each classifier on 3 feature sets: motifs alone (MOT), epigenetic marks alone (EPI) and both datasets combined (ALL). The detailed classification qualities measured by the Area Under the ROC Curve (AUC) in a10-fold cross-validation can be found in Table 1.

**Table 1:** Classification using different feature sets and classifiers

Dataset	BNFinder	SVM	RF
EPI	**0.9**	0.88	0.86
MOT	0.5	**0.89**	0.87
ALL	0.93	0.97	**0.98**

BNFinder seems to be indeed the best method for extracting the correct dependence of activity on the epigenetic marks, however it is unable to learn as much as other methods from datasets with more features. In particular, it fails completely on the motif-only feature set, indicating that there might not be a small subset of motifs allowing to predict activity. The other two methods perform similarly, although it should be noted that the random forest approach seems to be giving slightly, but statistically significantly (*p ≤ *10^−13 ^according to the model presented in [22]) better results in case of combined feature sets. Overall, all methods can improve significantly their accuracy by incorporating sequence information. In order to verify if the high predictive power of DNA motif information is not a product of a biased negative set or some very simple feature of enhancer sequences, we have performed two additional tests. Firstly, we have tested if the randomly chosen negative set is not biased towards low-complexity regions. For this purpose we have re-generated the negative sequence set avoiding the low-complexity regions annotated in the *Drosophila *genome. As can be seen in Table 2, such modified dataset gives almost the same classification results for both sets of features including histone marks indicating that the results were not biased by potentially poorer read-mapping efficiency in repeat regions. Interestingly, removing repeated regions makes the classification with motifs only almost as successful as with the complete feature set. This would be in line with the findings [23] that repeated regions such as transposons may harbor many transcription factor binding sites.

**Table 2:** Classification with repeat-masked negative sets

Dataset	SVM	RF
EPI	0.88	0.87
MOT	**0.96**	0.95
ALL	0.97	**0.98**

### Validating classifiers on known enhancers

In order to assess whether the classifiers might be useful to biologists, we wanted to go beyond standard cross-validation approaches and test the classifiers trained on the 8008 mesodermal enhancers on the more comprehensive, human-curated verified enhancer database. For this purpose we have used the well known Redfly database [24] that gathers enhancers reported in literature and makes them available with some human curation and additional annotation on tissue-specificity. As our training set was derived from mesodermal CRMs, we first tested our trained classifiers on the 250 enhancers reported to be active in mesoderm and then on 1480 enhancers non-specific to mesodermal tissue. Each dataset was complemented with a set of newly-generated random regions non-overlapping the training set (See Table S4, additional file 4 and S5, additional file 5).

The results are summarized in Table 2. It is clear that all classifiers give significantly non-random results. In the more predictable case of mesodermal enhancers, the complete feature-set expectedly gives the best performance. In case of non-mesodermal enhancers, however, the best performing classifier is the one based on sequence motif information. It seems that the performance of classifiers using epigenetic measurements specific to mesoderm was severely affected (in case of all enhancer up to to60%). However, the motif based classifier remained at a steady performance of 77%. This indicates that the motif information driving enhancer activity is possibly not as specific as it was thought earlier.

### Feature importance

In both partial sets the classification error is around 11-12 per cent, whereas in the ALL set the classification error drops significantly to 2 per cent. One should stress remarkable stability of the results. The average classification error in standard and reversed cross-validation scheme were very close to each other, in all cases the error is only 1 per cent higher in the reversed setup.

**Figure 2:**
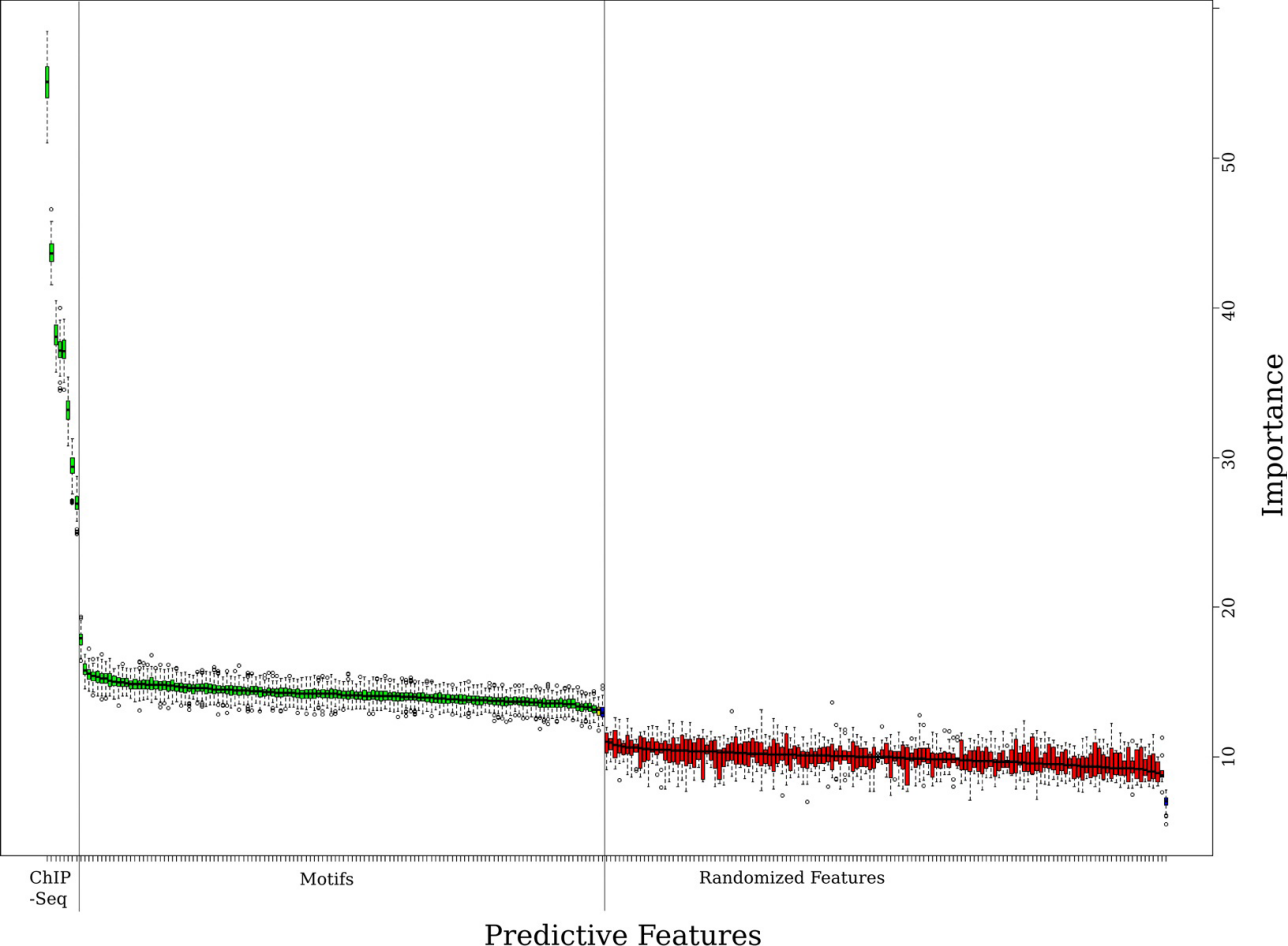
**Feature importance computed from Boruta package**. Relative importance of different features as computed by the Boruta package [29]. Each boxplot corresponds to a different feature and represents importance z-score from 500 randomizations. Histone modifications are the most important (z-score above 10), followed by all motif features (z-score above 3), all of which are separated from the randomized control variables with (red, z-scores below 3).

All features were identified as important by Boruta algorithm [25], in all three EPI, MOT and ALL data sets. In the ALL dataset the variables representing histone modifications were consistently ranked higher than those representing the motif binding, see Figure 2. (The detailed ranking of importance is given in Table S1, additional file 1).

The more detailed analysis of feature importance revealed several unexpected results. The iterative removal of least important TFs revealed that single transcription factor (zeste - TF 104 in our feature set) is sufficient to improve the classification accuracy to a level similar to that of the full classifier, see Table S2, additional file 2.

**Figure 3:**
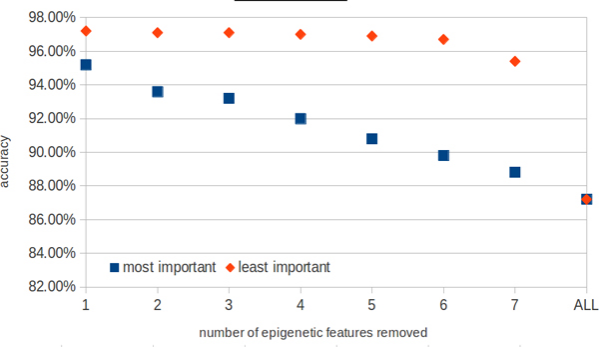
**Accuracy loss as a function of multiple chromatin feature removal**.

The analysis of redundancy between epigenetic modifications shows that removal of the most important modifications from the feature set leads to rapid degradation of the model quality. On the other hand removal of the least important modification decreases the model quality only gradually, see Figure 3.

**Figure 4:**
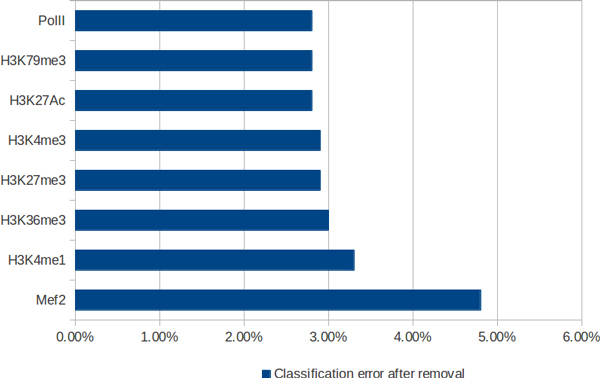
**Accuracy loss as a function of single chromatin feature removal**.

The analysis of these results suggests that a feature set consisting of single sequence motif (zeste) and three chromatin marks (H3K4me1, H3K36Me3 and Mef2 ChIP-Seq data) should be sufficient to build a model with stable prediction accuracy. This hypothesis was examined and it was confirmed by the 10-foldcross-validation. The average classification error obtained was 2.1 per cent. The quality of this model cannot be improved either by increasing the number of TFs or by adding more modifications. This is however not the only set of such features as removing any single motif (even the most important one) can be largely buffered by usage of the redundant information from the other features. In case of epigenetic marks, the situation is different, as removing any one of the three most important marks results in a significant loss of accuracy (see Figure 4). The most prominent marks are also the expected ones: Mef2 is a mesodermal transcription factor, H3K4Me1 is widely reported to be associated with enhancers and H3K36me3 is strongly correlated with transcribed regions, which are negatively correlated with regulatory activity.

## Conclusions

Our results strongly suggest that neither histone modifications nor sequence motif scan explain the total enhancer activity. However, our classification results for the complete data set are very promising suggesting that a model based on both types of features is sufficient to explain all phenomena represented in our training set. Relatively lower importance for motifs suggests that individual motifs are redundant - and cooperation of multiple TF is required. This is consistent both with our earlier results on purely sequence based prediction methods [9] as well as recent findings in heart-related enhancers [26]. The analysis of feature importance lead to the discovery of the reduced feature set comprising of three chromatin marks (Mef2+H3K4me1+H3K36me3) and a single transcription factor (TF 104 - zeste - a TF active in development) sufficient for a model with almost the same level of error that the full feature set. While all of these findings are in line with our current knowledge of the function of these features, it is difficult to make final biological conclusions due to redundancy between features. It is especially interesting in the context of apparent generality of the motif-based component of the classifier. Overall, our analysis proves that not only there is non-redundant information between motifs and epigenetic modifications, but we can show that it is enough to get near-perfect predictions of enhancer activity.

While our results are very promising, it should be noted that the training set is coming from a relatively simple model organism. In order to test if these findings can be applied to more complex systems such as mammalian genomes remains unanswered. While this question is vital for any medically oriented applications, it is currently very difficult to answer due to lack of comprehensive enhancer datasets such as Redfly [24]. Additionally, much larger size of mammalian genomes will undoubtedly be a challenge to computational scalability of machine learning methods.

## Methods

### Training enhancer sets

A small dataset, containing 23 positive and 39 negative samples was taken from work of Bonn et al. [16]. An average length of an enhancer was 1120 bp, maximum was 1985 bp and minimum was 999 bp.

A larger dataset containing 8008 samples of active enhancers was taken from the work of Zinzen et al. [17]. The average length of a positive sample in this set was 270.47 bp, maximum was 1182 bp and minimum 115 bp, with standard deviation of 112*bp*. The 8008 positive samples were complemented with an equal amount of randomly chosen negative samples. Negative set was chosen randomly from the remainder of the genome. Lengths of negative samples were chosen according to Gaussian distribution with the same mean and variance as observed in the positive set.

### Histone modification data

Histone modification ChIP-Seq data was taken from the work of Bonn et al. [16]. It contained 8 different chromatin marks:H3K4me1, H3K4me3, H3K27Ac, H3K27me3, H3K36me3, H3K79me3, Mef2, PolII, all measured between 6-8 h of development, values were given for windows of the length of 50 bp. The score for a given enhancer was averaged across all windows a sample overlapped with.

### Motif feature derivation

125 of used motifs were taken from the JASPAR database [18]. TRAP score [19] was used, in order to compute features based on the motifs. Parameters used while computing the TRAP score were left as default values of 0.7 for *λ *and *e*^0.584*·motif_length−*5.66 ^for *R*_0_.

### Classifier training

The Bayesian Network Classification was done with BNFinder [27], using Bayesian-Dirichlet equivalence (BDE) as the cost function. Because of computational cost of this method, cardinality of set of parents was limited to 3.

Used implementation of Random Forest comes from scikit-learn library for Python [28]. Classification was done using Random Forest Classifier, using 30 estimators.

Results obtained for the smaller dataset were generated in 4-cross-validation process. All the results presented for the larger dataset were averaged over 10-cross-validation folds. Subsets used forcross-validation were chosen randomly from shuffled samples.

### Enhancer datasets for validation

Validation of trained classifiers was performed on two enhancer datasets, coming from REDFly Database [24]. The general set contained 1830 samples of enhancers active for *Drosophila melanogaster*. The length of enhancers varied from 14 bp up to 22573 bp, with the averaging being 1829 kb.

The narrower, mesoderm related set consisted of 325 positive samples of enhancers annotated as active in mesodermal cells. The average length of marked enhancers was 1796 bp, maximal one was 20253 bp and minimal was 66 bp with standard deviation of 2285 bp.

Both positive sets were complemented with the equal amount of negative samples, chosen randomly from the remainder of the genome. Lengths of negative samples were chosen according to Gaussian distribution with the same mean and variance as observed in the positive set. In order to avoid bias in our favor, we have removed any regions overlapping the training set, decreasing the size of the larger training set to 1480 positive and 1824 negative samples. The smaller dataset was reduced to 250 positive and 325 negative samples.

### Feature importance

Assessment of feature importance and ranking was performed with the help of Boruta [29] library in R [30]. In this method the feature importance for classification obtained from Random Forest classifier [21] is compared between original feature and additional variables that do not carry information by design. The method is described in [25]. Boruta was used with default parameters. In the current paper additional procedure was applied to control the level of false positive discoveries. To this end the original system was extended by the set of contrast variables that don't contain information on decision variables by design - in the similar manner as within Boruta algorithm itself. The importance of variables in the set extended in this way was then examined using Boruta. The procedure was repeated 30 times with different realization of the contrast variables in each repetition. The average number of contrast variables that were deemed important by Boruta was a measure of expected number of false discoveries in the original set. The measure used for ranking the importance of features was a Z-score obtained from 30 steps of Boruta algorithm.

The procedure described above was applied to three datasets. In the first data set (MOD) the histone modifications were used as the descriptive variables and in the second set (MOT) the binding affinities for transcription factors from TRAP model. In the third set (ALL) both types were used.

We have performed additional analysis of importance of individual features for classification as well as extent of feature redundancy. The analysis was performed in a different way for histone modifications and for transcription factors. In all cases the starting point and reference set was the full feature set, containing all modifications and all transcription factors. In the case of modification we have examined the importance of individual features by removing the single modification from the feature set. We have also examined redundancy of information in modifications by removing *K *modifications at once, for *K *varying between one and seven. To keep number of tested combinations on a reasonable level the set of excluded modifications comprised either *K *most important modifications or *K *least important modifications. The number of features corresponding to transcription factors is much larger than the number of modifications and the importance of individual transcription factor is much smaller than importance of modifications. Therefore in the case of TFs the analysis was simpler. We applied an iterative procedure in which 80 per cent of least important TFs were removed from the information system examined in the previous step. When the number of TFs was smaller than 5 the single TF was removed.

### Classification with Random Forest

The classification was performed in two ten-fold cross-validation setups. In both setups the data was split in ten parts. In the first setup each 1/10th of the data set was once set aside as a test set and the remaining 9/10ths of the data set were used to train the classifier. Then the classification error was measured on the test set. The average error from all 10 test sets is then reported. In the second setup the role of the train and test set are reversed - the classifier is trained using 1/10th of the data set and the error is measured using the remaining 9/10ths of the data.

## Competing interests

The authors declare that they have no competing interests.

## Authors' contributions

BW designed the study; AP prepared all datasets and performed initial classifier training with BNfinder, SVMs and RFs as well as the functional validation against the Redfly database. Final RF training and feature importance measurements with Boruta package was performed by MW, WP and WR. BW drafted the manuscript based on contributions from all authors.

**Table 3:** Validation of classifiers on the Redfly database

Dataset	Redfly Meso	RedFly
EPI	0.77	0.62
MOT	0.74	**0.77**
ALL	**0.78**	0.75

**Table 4:** Classification quality with different cross- validation schemes

Dataset	Cross-validation 9:1	Cross-validation 1:9
EPI	88.2 ± 0.6%	87.3 ± 0.2%
MOT	89.9 ± 0.9%	87.2 ± 0.6%
ALL	98.1 ± 0.5%	97.2 ± 0.4%

## Supplementary Material

Additional file 1 **Table S1 -- Detailed ranking of feature importance**. For the convenience of the reader, all supplementary information can also be obtained from the supplementary website http://bioputer.mimuw.edu.pl/papers/enhancer_prediction. Click here for file

Additional file 2 **Table S2 -- Results of iterative feature removal**. For the convenience of the reader, all supplementary information can also be obtained from the supplementary website http://bioputer.mimuw.edu.pl/papers/enhancer_prediction. Click here for file

Additional file 3 **Table S3 -- Training set**. For the convenience of the reader, all supplementary information can also be obtained from the supplementary website http://bioputer.mimuw.edu.pl/papers/enhancer_prediction. Click here for file

Additional file 4 **Table S4 -- Redfly mesodermal testing set**. For the convenience of the reader, all supplementary information can also be obtained from the supplementary website http://bioputer.mimuw.edu.pl/papers/enhancer_prediction. Click here for file

Additional file 5 **Table S5 -- Redfly non-specific testing set**. For the convenience of the reader, all supplementary information can also be obtained from the supplementary website http://bioputer.mimuw.edu.pl/papers/enhancer_prediction. Click here for file

## Declaration

The publication fee was covered in full by the National Center for Science grant decision number DEC-2012/05/B/N22/0567.

This article has been published as part of *BMC Systems Biology *Volume 7 Supplement 6, 2013: Selected articles from the 24th International Conference on Genome Informatics (GIW2013). The full contents of the supplement are available online at http://www.biomedcentral.com/bmcsystbiol/supplements/7/S6.
