# Supplementary material for: Active enhancer positions can be accurately predicted from chromatin marks and collective sequence motif data
Source: BMC Syst Biol. 2013 Dec 13;7(Suppl 6):S16. doi: 10.1186/1752-0509-7-S6-S16 (PMC4029456; doi:10.1186/1752-0509-7-S6-S16)
Supplement: Additional file 1 — Table S1 -- Detailed ranking of feature importance. For the convenience of the reader, all supplementary information can also be obtained from the supplementary website http://bioputer.mimuw.edu.pl/papers/enhancer_prediction. [file 1752-0509-7-S6-S16-S1.PDF]

Supplementary Table 1

Importance of all features in random forest, features are sorted according to mean Zscore. Average, minimal and maximal Zscore recorded in Boruta run are presented, as well as fraction of random forest runs where the importance of the feature was higher than maximal importance of the contrast variable.

Var	Z_{mea}	Z_{min}	Z_{max}	frac
**G**	51,1	48,0	54,7	1,00
**D**	36,5	33,5	39,9	1,00
**C**	30,1	27,4	33,1	1,00
**A**	29,3	26,7	32,1	1,00
**H**	28,8	26,3	31,2	1,00
**B**	24,4	22,3	26,2	1,00
**E**	20,6	18,4	23,4	1,00
**F**	17,8	16,0	19,3	1,00
**V104**	8,7	7,5	10,2	1,00
**V6**	6,5	5,4	7,9	1,00
**V59**	6,2	5,0	7,7	1,00
**V82**	6,1	5,2	7,4	1,00
**V86**	6,0	4,9	7,1	1,00
**V10**	5,9	4,6	7,2	1,00
**V53**	5,9	4,4	7,0	1,00
**V19**	5,8	4,6	7,2	1,00
**V13**	5,6	4,8	6,6	1,00
**V81**	5,5	4,4	6,4	1,00
**V88**	5,5	4,4	6,6	1,00
**V48**	5,4	4,6	6,7	1,00
**V66**	5,4	4,2	6,6	1,00
**V17**	5,4	4,3	6,5	1,00
**V4**	5,4	4,3	6,6	1,00
**V38**	5,4	4,5	6,5	1,00
**V109**	5,4	4,0	6,7	1,00
**V78**	5,3	4,2	6,6	1,00
**V34**	5,3	4,3	6,2	1,00
**V62**	5,3	3,8	6,4	1,00
**V9**	5,2	3,9	6,1	1,00
**V98**	5,1	4,2	6,1	1,00
**V123**	5,1	3,7	6,2	1,00
**V31**	5,1	4,0	6,1	1,00
**V1**	5,1	4,1	6,0	1,00
**V122**	5,0	3,7	6,8	1,00
**V42**	5,0	3,8	6,1	0,98
**V87**	5,0	4,1	6,4	0,98
**V3**	5,0	3,8	6,1	1,00
**V20**	5,0	4,0	6,5	1,00
**V101**	5,0	4,2	6,0	1,00
**V113**	5,0	4,1	6,1	1,00
**V23**	5,0	4,0	6,1	0,98
**V69**	4,9	4,3	5,9	1,00
**V37**	4,9	3,8	6,2	1,00
**V103**	4,9	3,8	6,0	1,00
**V96**	4,9	3,9	5,9	1,00

Var	Z_{mea}	Z_{min}	Z_{max}	frac
**V18**	4,9	3,6	6,1	0,98
**V97**	4,8	3,7	6,0	0,98
**V65**	4,8	3,7	6,0	1,00
**V60**	4,8	3,7	5,9	1,00
**V63**	4,8	4,0	5,8	1,00
**V80**	4,8	3,7	6,0	1,00
**V125**	4,8	3,7	5,7	0,98
**V56**	4,8	3,9	5,8	0,98
**V76**	4,7	3,7	5,8	0,98
**V67**	4,7	3,9	5,6	0,98
**V12**	4,7	3,8	5,9	0,98
**V106**	4,7	3,6	5,7	1,00
**V28**	4,7	3,4	5,6	0,97
**V44**	4,6	3,9	5,7	0,98
**V41**	4,6	3,6	5,9	1,00
**V68**	4,6	3,8	5,2	0,98
**V93**	4,6	3,6	5,6	1,00
**V46**	4,6	3,4	6,0	1,00
**V2**	4,6	3,5	5,3	0,98
**V50**	4,5	3,7	5,5	0,97
**V120**	4,5	3,6	5,4	1,00
**V22**	4,5	3,7	5,6	1,00
**V84**	4,5	3,2	5,8	0,97
**V29**	4,5	3,4	5,4	0,98
**V95**	4,5	3,4	5,8	0,98
**V92**	4,5	3,4	6,1	1,00
**V71**	4,5	3,4	5,5	1,00
**V47**	4,5	3,4	5,4	0,98
**V83**	4,4	3,4	5,4	0,98
**V99**	4,4	3,6	5,2	0,97
**V102**	4,4	3,3	5,4	0,97
**V75**	4,4	3,3	5,5	1,00
**V91**	4,4	3,3	5,5	0,97
**V105**	4,4	3,3	5,6	0,98
**V39**	4,4	3,6	5,4	0,98
**V117**	4,3	3,4	5,3	0,97
**V61**	4,3	3,4	5,3	1,00
**V24**	4,3	2,8	5,3	1,00
**V30**	4,3	3,2	5,4	0,97
**V94**	4,3	3,4	5,2	0,95
**V57**	4,2	3,2	5,7	0,98
**V100**	4,2	3,3	5,2	0,97
**V52**	4,2	3,0	5,2	0,97
**V70**	4,2	3,1	5,0	0,97

Var	Z_{mea}	Z_{min}	Z_{max}	frac
**V16**	4,2	3,3	5,2	0,98
**V14**	4,2	3,0	5,3	0,95
**V7**	4,1	3,2	5,1	0,96
**V5**	4,1	3,2	5,2	0,97
**V64**	4,1	3,2	5,1	0,96
**V85**	4,1	3,2	5,0	0,96
**V74**	4,1	2,9	5,4	0,96
**V8**	4,1	3,4	5,3	0,97
**V111**	4,1	3,1	5,3	0,96
**V79**	4,0	3,1	5,1	0,96
**V26**	4,0	3,0	5,0	0,96
**V32**	4,0	2,9	5,1	0,95
**V45**	4,0	3,2	4,9	0,94
**V25**	4,0	3,2	5,1	0,98
**V54**	4,0	2,8	5,2	0,92
**V40**	4,0	3,1	5,1	0,95
**V36**	4,0	2,7	4,8	0,95
**V15**	3,9	2,8	4,9	0,94
**V77**	3,9	2,7	5,0	0,91
**V124**	3,9	3,0	4,6	0,94
**V55**	3,9	2,9	4,6	0,93
**V27**	3,9	3,0	4,6	0,94
**V89**	3,9	2,8	4,8	0,96
**V35**	3,8	2,9	4,6	0,93
**V107**	3,8	2,8	4,9	0,94
**V21**	3,8	3,1	4,9	0,92
**V43**	3,8	2,8	4,9	0,94
**V49**	3,7	2,6	4,7	0,95
**V119**	3,7	2,8	4,8	0,92
**V73**	3,7	2,9	4,8	0,93
**V110**	3,7	2,7	4,7	0,93
**V58**	3,7	3,0	4,6	0,94
**V72**	3,7	2,9	4,9	0,94
**V118**	3,7	2,7	4,8	0,89
**V51**	3,7	2,6	4,7	0,92
**V90**	3,6	2,9	4,8	0,91
**V112**	3,6	2,4	4,7	0,89
**V33**	3,6	2,8	4,3	0,91
**V108**	3,4	2,2	4,2	0,89
**V116**	3,4	2,5	4,5	0,87
**V114**	3,4	2,4	4,2	0,88
**V11**	3,3	2,4	4,2	0,82
**V115**	3,2	2,0	4,5	0,79
**V121**	3,1	1,9	4,1	0,66
