# Supplementary material for: Active enhancer positions can be accurately predicted from chromatin marks and collective sequence motif data
Source: BMC Syst Biol. 2013 Dec 13;7(Suppl 6):S16. doi: 10.1186/1752-0509-7-S6-S16 (PMC4029456; doi:10.1186/1752-0509-7-S6-S16)
Supplement: Additional file 2 — Table S2 -- Results of iterative feature removal. For the convenience of the reader, all supplementary information can also be obtained from the supplementary website http://bioputer.mimuw.edu.pl/papers/enhancer_prediction. [file 1752-0509-7-S6-S16-S2.PDF]

Supplementary Table 2a

Classification results for reduced sets comprising all modifications and and K most important TFs

Classification results

Number of Tfs in the feature set	Classification error
125	2,7%
100	2,6%
80	2,4%
64	2,4%
52	2,3%
42	2,3%
34	2,2%
27	2,2%
22	2,1%
18	2,1%
15	2,1%
12	2,0%
10	2,0%
8	2,1%
7	2,1%
6	2,1%
5	2,1%
4	2,1%
3	2,0%
2	2,0%
1	2,1%

The inspection of the influence of individual modifications reveals that three modifications are non-redundant – their removal from the feature set results in increase of classification error, see Supplementary Table 2b.

Supplementary Table 2b

Classification results for reduced sets comprising all TFs and all but one ribosome modification

Modification removed from the feature set	Classification error
A	2,8%
B	2,9%
C	3,0%
D	3,3%
E	2,9%
F	2,8%
G	4,8%
H	2,8%

The analysis of redundancy between ribosomal modifications shows that removal of the most important modifications from the feature set leads to rapid degradation of the model quality, see Supplementary Table 2b.
